# Orchestrating life’s first community: molecular assembly by human milk oligosaccharides

**DOI:** 10.1080/19490976.2026.2632973

**Published:** 2026-02-19

**Authors:** Ye Zhou, Bo Yang, Jianxin Zhao, Paul Ross, Catherine Stanton, Wei Chen

**Affiliations:** a State Key Laboratory of Food Science and Resources, Jiangnan University, Wuxi, Jiangsu, People's Republic of China; b School of Food Science and Technology, Jiangnan University, Wuxi, Jiangsu, People's Republic of China; c International Joint Research Laboratory for Maternal-Infant Microbiota and Health, Jiangnan University, Wuxi, Jiangsu, People's Republic of China; d APC Microbiome Ireland, University College Cork, Cork, Ireland; e Teagasc Food Research Centre, Moorepark, Fermoy, Cork, Ireland

**Keywords:** Human milk oligosaccharides, infant gut microbiota, microbial assembly, molecular mechanisms, prebiotic substrate, signaling molecules

## Abstract

The infant gut microbiota, orchestrated by human milk oligosaccharides (HMOs), forms a critical foundation for lifelong health. Despite their recognized importance, the molecular strategies through which HMOs govern microbial competition and niche establishment remain poorly understood. Moving beyond ecological observations, this review synthesizes current mechanistic evidence on the molecular machinery of HMO metabolism in microbial assembly. We explore the specialized enzymes that confer competitive advantages and the metabolic networks fueled by HMO breakdown. Furthermore, we distinguish substrate-driven effects from the hypothesized signaling roles of intact HMOs in modulating host-microbe interactions, indicating where the evidence is associative versus causal. By integrating these pathways, we provide a blueprint for leveraging HMO biology to develop targeted nutritional interventions for preventing early-life disorders.

## Graphical abstract

**Figure:**
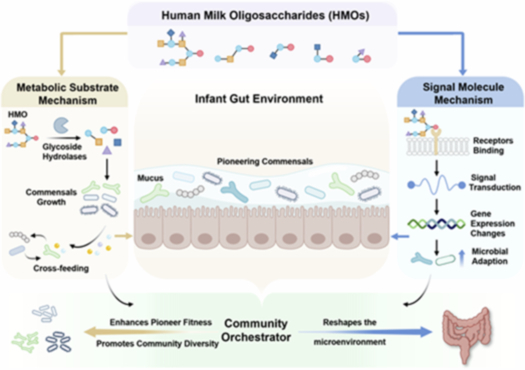

## 1. Introduction

The infant gut is a highly dynamic ecosystem, and its initial microbial colonization constitutes a critical developmental window. The microbiota established during this period profoundly and durably impacts host metabolism, immune function, and long-term health.^1^^,^^2^ Perturbations to this succession, such as dysbiosis due to cesarean birth, antibiotic exposure, or nutritional deficits, are linked to increased susceptibility to various diseases.^3^^,^^4^ In recent years, extensive research has explored the successional dynamics of the infant gut microbiota, spanning broad compositional characteristics^5^ and strain-level variation.^6^ It is now established that early-life colonization patterns are far from stochastic; rather, they are shaped through the interaction of ecological and environmental forces with early colonizers seeded from diverse sources.

Human milk oligosaccharides (HMOs) act as a fundamental regulator in shaping the developing gut microbiome. Their primacy stems from serving a role as the main carbon and energy source for the early-life colonic microbiota. The imperative to access this limited resource in the infant gut is a key determinant of microbial colonization success, a principle demonstrated by studies showing how carbon availability dictates gut occupancy by commensals such as *Bacteroides thetaiotaomicron.*^7^^,^^8^ HMOs are uniquely equipped to impose selective driver of colonization. Resistance to host digestion ensures the intact delivery of HMOs to the colon, while complex structural diversity necessitates specialized genetic machinery for catabolism. By creating a nutrient niche, HMOs drive the sustainable colonization of bacterial taxa harboring genetic adaptations for their utilization, particularly *Bifidobacterium*^9^ and *Bacteroides* species.^10^ This selective enrichment, and the subsequent metabolites released from HMOs, foster complex cross-feeding networks that underpin the structure and stability of the broader microbial community.^11^

The role of HMOs in shaping the development and assembly of the infant gut microbiome is well-established.^12^ Extensive *in vitro* and *in vivo* evidence, as systematically summarized by Jackson et al., indicates that HMOs selectively enrich gut microbial taxa capable of their degradation, most notably *Bifidobacterium* and *Bacteroides*, while reducing the abundance of potential pathogens such as *Enterobacteriaceae*. This selective modulation thereby shapes a microbial community structure associated with healthier infant development.^13^ However, current understanding remains largely descriptive, relying predominantly on correlative observations rather than mechanistic explanations. A fundamental question thus remains unsolved: how do specific HMOs mechanistically determine bacterial fitness and colonization success? This knowledge gap is further widened by the prevailing research focus on HMOs as metabolic substrates, while overlooking the biological significance of the substantial fraction that escapes degradation through the infant colon.^14^

With the maturation of high-resolution multi-omics,^15^ functional genomics^16^ and advanced *in vitro* models,^17^ research emphasis has turned from associative observations to deciphering the molecular mechanisms underpinning HMO-gut microbe interactions. These technical advances have revealed that genetic determinants essential for HMO utilization dictate microbial fitness and niche partitioning in the infant gut,^18^ with the underlying enzymes exhibiting high species-specificity. Furthermore, metabolites derived from HMO mediate ecological interactions via nutrient competition and cross-feeding.^19^ Such interactions, in turn, support key community-level properties including microbial assembly,^20^ stability,^21^ and colonization resistance.^22^ Beyond this metabolic role, HMOs engage host pattern recognition receptors and trigger a cascade of effects—such as impaired pathogen adhesion, altered glycan glycosylation, and immunoregulation—collectively shaping a host microenvironment favorable to microbial colonization.^23–25^ This establishes the role of HMOs as signaling molecules beyond nutrition. This review synthesizes how pioneer bacteria exploit HMOs to establish and sustain a durable gut niche, focusing on the molecular mechanisms by which HMOs act as both prebiotic nutrients and non-metabolic signaling molecules in shaping the infant gut ecosystem.

## 2. The dynamic assembly of infant gut microbiota: succession and ecological drivers

The establishment and maturation of the infant gut microbiome follow a successional trajectory. Although the timing of initial bacterial colonization— whether *in utero* or at birth— remains debated, birth unequivocally represents a critical window for microbial inoculation, introducing a substantial diversity of microorganisms from maternal and environmental sources.^6^ While the initial microbial inoculum is highly stochastic, its subsequent succession is shaped by the interplay of four key ecological processes: dispersal (the immigration of new microbes), selection (the deterministic shaping of communities by host and environmental filters), drift (stochastic changes in population sizes), and diversification (the generation of genetic or phenotypic variation within populations).^26^^,^^27^ Among these, selection is paramount. Selective forces act on early colonizers throughout succession, shaping the initial diversity into a developmentally specific pioneer community.

Selective forces operate within a hierarchical framework, wherein higher-order factors constrain the effects of lower-level interactions. This concept is reflected in emerging theoretical models proposing a hierarchical structure for microbial assembly with host-mediated habitat filtering exerting primary selective pressure.^28^ Shortly after birth, neonates acquire a diverse range of maternal and environmental microbes. Taxa such as *Staphylococcus*, *Streptococcus*, and *Lactobacillus* are commonly detected in meconium.^29^^,^^30^ This initial microbial inoculum is rapidly refined through habitat filtering driven by host-derived factors, including pH, oxygen levels, nutrient availability, and immune activity.^31^ Under these constraints, the signature taxa in the neonate gut shift from facultative anaerobes (e.g., *Enterobacteriaceae*) to obligate anaerobes such as *Bifidobacterium* and *Bacteroides* within the first few weeks. This resulting community is further selected by host dietary inputs, as evidenced by the distinct microbiota profiles observed in breastfed versus formula-fed infants.^32^^,^^33^ In addition, microbiota-intrinsic interactions, such as priority effects, play a critical role in structuring the community. By modifying or preempting ecological niches, these effects determine whether incoming strains can replace established colonizers or are excluded, thus stabilizing the assembled population while continually influencing its dynamics. A notable example is *B. breve*, which can achieve dominance via niche-preemptive colonization despite its relatively limited capacity for HMO utilization.^34^ In summary, the assembly of the infant gut microbiome is governed by a succession of host-derived and microbiota-intrinsic selective pressures. Pioneer bacteria that ultimately persist are those whose fitness allows them to successfully pass through this hierarchical series of filters.

## 3. HMOs as central drivers of microbial assembly

HMOs function as foundational drivers of gut microbial assembly, a role rooted in their unique structural properties and high degree of tunability. Structurally, HMOs exhibited a diverse and intricate architecture based on heterogeneously branched lactose or *N*-acetyl-lactosamine units, frequently modified with fucose and sialic acid.^35^ This structural specificity distinguishes HMOs from broad-spectrum prebiotics such as fructo-oligosaccharides/galacto-oligosaccharides (FOS/GOS), which can be utilized by potential pathobionts. Consequently, HMOs are classified as targeted prebiotics that selectively enrich taxa equipped with the corresponding genomic repertoire, enabling precise microbial niche engineering within the developing gut.^36^ Moreover, unlike innate selective forces such as habitat filtering, the effects of HMOs on the gut microbiota are highly tunable. Efficient synthesis techniques now enable the large-scale production of specific HMOs, making this tunability practicable.^37^ Regulatory approvals by major agencies (e.g., GRAS in the US, EFSA in the EU) permit the inclusion of defined HMOs, such as 2′-FL and LNnT, in infant formula at typical doses of 0.1–2 g/L. Clinical trials of formulas containing these HMOs report bifidogenic shifts, elevated fecal sIgA, and a reduced incidence of certain gastrointestinal infections over 3–6 months;^38^^,^^39^ however, evidence pertaining to long-term health outcomes remains limited. Collectively, these attributes establish HMOs as a deterministic and potent selective force in early-life microbiome assembly.

Experimental evidence from model systems and human cohorts establishes HMOs as drivers that override initial stochastic colonization to ensure a predictable successional trajectory. For instance, a longitudinal study tracking microbial dynamics from birth to six months revealed that *B. longum* subsp. *infantis*, an optimal HMO utilizer, consistently outcompeted earlier colonizers such as *B. catenulatum* and *Bacteroides* species to achieve dominance.^40^ The strength of this HMO-driven selection is sufficient to overcome colonization resistance in even the adult microbiome, permitting reversible engraftment and steady-state abundance of *B. infantis.*^41^^,^^42^ This suggests that the privileged niche created by HMOs provides a mechanistic rationale for targeted microbial manipulation, guiding the design of synbiotic interventions to enforce stable, beneficial consortia. Furthermore, HMOs selectively enrich a broad spectrum of utilizers beyond bifidobacteria, including *Bacteroides*, *Akkermansia*, *Roseburia*, and *Streptococcus.*^43–45^ The prevalence of these taxa in breastfed infants, coupled with their stimulation by specific HMOs,^46^^,^^47^ highlights the HMO-driven assembly of a multi-kingdom consortium specialized in their catabolism. It is worth noting that HMO-driven microbiome assembly is not a simple sum of individual glycan-species interactions, but a complex trophic network shaped by interspecies resource competition and facilitation. The well-documented cross-feeding among bifidobacteria not only enables efficient HMO utilization but also enhances niche establishment, underpinning their collective dominance in the infant gut.^48^

## 4. Molecular mechanisms of HMO-driven gut microbiota assembly

**Figure 1.**
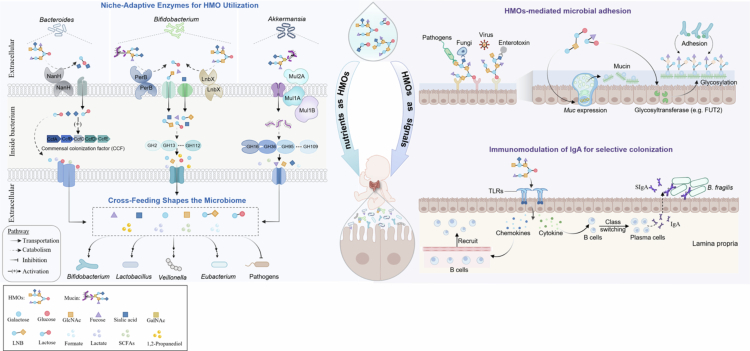
Molecular mechanisms underlying HMO-mediated assembly of the infant gut microbiota. As metabolic substrates, HMOs are degraded by pioneering commensals (e.g., *Bacteroides*, *Bifidobacterium*, *Akkermansia*) using specialized enzymes and transporters, providing them with a competitive advantage. The resulting metabolites fuel a cross-feeding network that supports the growth of other beneficial commensals (e.g., *Lactobacillus*, *Veillonella*, *Eubacterium*), thereby driving microbial community assembly. As signaling molecules, intact HMOs act as soluble decoys to inhibit pathogen adhesion while enhancing commensal adhesion by modulating mucosal glycosylation. Furthermore, HMOs interact with host pattern-recognition receptors to shape immune responses, such as regulating sIgA production, which in turn selects for specific microbial colonization.

Moving beyond phenomenological observations, recent advances in multi-omics and functional genomics empower a mechanistic dissection of how HMOs govern microbial assembly. Here, we delve into the molecular specifics. We first examine HMOs as metabolic substrates, whose degradation by specialized enzymatic machinery enables niche competition, while the liberated metabolites orchestrate community assembly. We then explore how HMOs function as signaling molecules, directly modulating microbial adhesion and host immunity to forge a symbiont-promotive niche (Figure 1).

### 4.1. Metabolic regulation by HMOs

#### 4.1.1. Enzymatic adaptation drives niche competition for HMOs

In the nutrient-limited infant gut, pioneering commensals gain a competitive advantage through specialized HMO-degrading enzymes, a key adaptation for privatizing this restricted carbon niche. Although these enzymatic systems (e.g., glycoside hydrolases and transporters) are well-characterized,^35^ their ecological significance in promoting microbial persistence and competitive dominance remains inadequately defined. Compared to *Bifidobacterium*, the advancement of gene editing technologies in *Bacteroidetes*, particularly through CRISPR-Cas-based tools, enables a more in-depth exploration of the molecular mechanisms governing *Bacteroidetes*-HMOs-host interactions.^43^ Recent studies combining gene knockout with *in vivo* colonization models have shown that NanH, a sialidase encoded within the HMO PUL1 locus of *Bacteroides fragilis*, is essential for the degradation of both host mucins and sialylated HMOs. Critically, NanH-dependent catabolism endows *B. fragilis* with enhanced ecological fitness, as demonstrated by the superior colonization persistence and niche resilience of *nanH*-harboring strains that consistently outcompete *nanH* knock-out mutants in defined microbial communities and rapidly reestablish gut dominance following antibiotic perturbation.^49^ These findings, primarily derived from gnotobiotic or defined community models in mice, identify NanH sialidase as a key facilitator of commensal colonization in early life. Its strain specificity, substrate preferences, and translational relevance to the complex, diverse infant gut ecosystem warrant further investigation. Similarly, *B. bifidum* employs the extracellular sialidase SiaBb2 to mediate mucosal adhesion by specifically recognizing α2,6-linked sialyloligosaccharides and blood group A antigens on mucins.^50^ While *in vitro* adhesion assays support this mechanism, direct evidence for SiaBb2's function in *in vivo* colonization remains to be fully elucidated. The extensive intestinal surface sialylation, neonatal enrichment of bacterial sialidases, and their phylogenetic conservation confer sialidases' dominant regulatory role in early-life microbiota assembly compared to other HMO-degrading enzymes.^51^^,^^52^ Notably, pathogenic bacteria also harbor sialidases encoded by Nan cluster, yet deploy them to expose specific mucosal adhesin and toxin receptors, facilitating invasive colonization and pathogenesis. For instance, sialidases NanH, NanJ and NanI in *Clostridium perfringens* are crucial for mucin degradation and mucosal colonization.^53^ Despite genomic conservation, substrate preferences of Nan sialidases differ between commensals and pathogens: CpNanI (*C. perfringens*) and HpNanH (*Glaesserella parasuis*) preferentially target Siaα2-6Gal linkages, whereas BbSia2 (*B. bifidum*) specifically recognizes Siaα2-6GlcNAc.^54^ These distinct specificities critically influence gut colonization patterns, suggesting that selective sialidase inhibitors or prebiotic-based modulation could enable precise manipulation of microbial community assembly.

While sialidases facilitate colonization through acidic HMOs, fucosylated glycans provide complementary nutrients that further support microbial establishment. The fucosylated niche is fundamentally shaped by Fut2-mediated α1,2-fucosylation, which modifies both secreted HMOs and host membrane glycoconjugates to regulate microbial access to L-fucose residues.^55^ Longitudinal cohort studies consistently show enriched *Bifidobacterium* populations in breastfed infants of secretor mothers, underscoring the ecological significance of fucosylated HMOs.^56^ Regarding the underlying mechanisms, although ABC transporters such as FL-SBP were initially proposed to mediate bifidobacterial colonization via fucosylated HMO uptake, conclusive experimental evidence remains fragmentary - limited primarily to *in vitro* studies showing impaired HMO utilization in FL-SBP knockout mutants.^18^ In contrast, growing evidence identifies the enzymatic utilization of fucose liberated from dietary or host glycans as a principal determinant of successful gut colonization. For instance, *E. coli* mutants lacking *fucK* and *fucAO* show impaired intestinal persistence despite initial colonization capacity.^57^ Furthermore, *Bacteroides* species can incorporate exogenous fucose directly into fucosylated glycoproteins; deletion of *lfg* disrupts this protein glycosylation, which is essential for competitive colonization by *Bacteroides.*^58^ These findings establish HMO cross-feeding as a fundamental driver of gut ecosystem assembly, where cooperative metabolic networks facilitate microbial co-existence while inherently promoting ecological niche partitioning through substrate specialization.

In addition to the niche specialization conferred by fucosylated HMO-degrading enzymes, glycosidases of the GH136 family critically drive the establishment of distinct microbial niches through the catabolism of neutral HMOs. The GH136 family was first defined upon the identification of LnbX, a novel lacto-*N*-biosidase (LNBase) that cleaves lacto-*N*-biose from lacto-*N*-tetraose (LNT).^59^ Although functionally similar to the GH20 lacto-*N*-biosidase (LnbB), LnbX differs significantly in amino acid sequence, structural architecture, and substrate preference. Genetic evidence confirms that LnbX is essential for *Bifidobacterium longum* growth on LNT and constitutes a key genetic adaptation for its persistence in the gut of breastfed infants.^59^ Extending the study of GH136 enzymes in host adaption, recent multi-omics studies integrating rodent colonization models and targeted gene editing have demonstrated that the *perB* gene, encoding a GH136 family glycosyl hydrolase in *Bifidobacterium*, acts as a strain-specific determinant enabling sustained gut colonization from infancy to adulthood.^60^ Although PerB is primarily implicated in mucin glycan degradation, its enzymatic activity may also target HMOs due to structural similarities between mucin glycans and HMOs.^61^ Notably, PerB-associated colonization has been observed to exhibit a female-predominant pattern in adult and maternal contexts.^60^ Given that sex hormone activity is minimal in early infancy, a key unresolved question is the extent to which PerB-mediated utilization of HMOs contributes to colonization dynamics in infants.

Among the diverse enzymatic systems capable of degrading HMOs and mucins, polysaccharide utilization loci (PULs) stand out as a distinct mechanism of glycans utilization and gut adaption for *Bacteroidetes*. These conserved gene clusters typically encode glycolytic enzymes, glycan-binding proteins, and transcriptional regulators, enabling the targeted catabolism of complex dietary and host-derived glycans.^62^ Critically, PUL-mediated glycan utilization extends beyond metabolic adaptation to serving as a deterministic driver of microbial colonization dynamics. Pivotal early studies in *Bacteroides thetaiotaomicron* demonstrated that disrupting five transcriptional regulators suppresses O-glycan-responsive PUL expression, compromising bacterial persistence in the gut niche.^62^ These mutants consistently exhibit competitive fitness defects against wild-type strains during vertical transmission, underscoring the indispensable role of PULs in mother-to-offspring microbial colonization. Beyond transcriptional regulation, a sophisticated post-transcriptional network—centered on the global RNA-binding protein RbpB and a conserved family of paralogous small RNAs (sRNAs)—further orchestrates PUL translation in *Bacteroides.*^63^ This hierarchical regulation enables dynamic substrate prioritization by repressing redundant PULs, conferring a colonization advantage in the gut ecosystem. Despite well-characterized regulatory networks, the specific enzymatic components underlying PUL-mediated niche colonization remain largely uncharacterized. A notable exception is the commensal colonization factor (CCF) locus (*ccfABCDE*)—a unique subset of PULs molecularly demonstrated to mediate species-specific saturable colonization.^64^ Functionally, the CCF system confers conspecific colonization resistance through competitive nutrient exclusion, while enabling *Bacteroides fragilis* to penetrate and stably occupy colonic crypts, thereby promoting cross-species persistence. Furthermore, CCF is required for *B. fragilis* to reestablish colonization after microbiome perturbation, whether induced by *Citrobacter rodentium* infection or antibiotic exposure. This highlights its critical role in early-life microbiota assembly, particularly given that the immature gut ecosystem is susceptible to ecological drift that may disrupt normal successional trajectories.

Parallel to the PULs in *Bacteroidetes*, the mucin utilization locus (MUL) represents a specialized genetic system that endows *Akkermansia muciniphila* with the capacity to degrade and assimilate mucins, thereby reinforcing its mucosal colonization advantage. As a promising next-generation probiotic, *A. muciniphila* demonstrates exceptional efficiency in mucin catabolism, through a dedicated set of carbohydrate-active enzymes that systematically break down mucin O-glycans.^65^ Nevertheless, the mechanistic link between mucin utilization and colonization of *Akkermansia* remained elusive, largely due to its genetic intractability. Recent breakthroughs in transposon mutagenesis coupled with insertion sequencing have addressed this persistent challenge, demonstrating that the MUL—encoding pili and a periplasmic protein complex—is essential for outer membrane mucin transport and, consequently, stable gut colonization.^66^ Notably, the functional principles of *A. muciniphila*'s MUL differ significantly from the canonical PUL systems in *Bacteroides*. Whereas PULs are essential for *Bacteroides* colonization regardless of microbial competition,^64^ MUL-facilitated colonization confers a competitive edge to *A. muciniphila* specifically within polymicrobial gut ecosystems. This ecological specialization aligns with their regulatory differences: *Bacteroides* PULs are substrate-inducible, while MUL genes are constitutively expressed independent of mucin. Such constitutive MUL activation likely evolved to support sustained mucosal colonization by *A. muciniphila*, contrasting with the nutrient-dependent paradigm of *Bacteroides*.

Collectively, these studies establish that a defined repertoire of enzymatic systems constitutes the molecular basis for glycan-driven niche competition (Table 1). However, our current mechanistic understanding is largely based on reductionist murine models and exhibits species- and strain-specificity. Translating this mechanistic map to the human infant gut requires resolving key unknowns: the functional contribution of these systems *in vivo*, their regulatory interplay, and how their activity is modulated by host and dietary factors (e.g., community composition, glycan profiles). The diversity of these enzymatic strategies illustrates the adaptive radiation of pioneer taxa under HMO selection pressure, underscoring the need for future research in ecologically relevant human contexts.

**Table 1.** Enzymatic systems for HMO and mucin glycan utilization in early-life gut colonization.

Module	Representative taxa	Key genes/enzymes/loci	Substrate(s)	Ecological effect	Evidence tier
Sialidase-mediated utilization	*Bacteroides fragilis, B. bifidum*	*nanH,* SiaBb2	Sialylated HMOs, mucins	Competitive colonization, niche resilience	*In vitro,* Animal models
Fucose utilization systems	*Bacteroides* spp., *Escherichia coli*	*fuc* clusters (*fucK*, *fucAO*), *lfg*	Fucose liberated from fucosylated HMOs or host glycans	Colonization persistence, glycoprotein fucosylation	*In vitro*, Animal models
GH136 family lacto-*N*-biosidases	*Bifidobacterium longum*, *B. bifidum*	*lnbX*, *perB*	Neutral HMOs (e.g., LNT), mucin glycans	Sustained colonization	*In vitro*, Animal models
Polysaccharide Utilization Loci (PULs)	*Bacteroides thetaiotaomicron*, *Bacteroides fragilis*	RbpB, sRNAs, *ccfABCDE locus*	Host glycans	Competitive fitness, niche resilience	*In vitro*, Animal models
Mucin Utilization Locus (MUL)	*Akkermansia muciniphila*	MUL	Mucins	Mucosal colonization, competitive advantage	Animal models

#### 4.1.2. Metabolites orchestrate ecological networking

Given that direct HMO degradation is restricted to specific taxa, a central question remains: how HMOs promote the establishment of a diverse and stable infant gut microbiota? HMO-derived metabolites, specifically Short-chain fatty acids (SCFAs), display developmental stage-specific profiles in the infant gut. For instance, fucose-derived formate has been shown to positively correlate with bifidobacterial colonization.^67^ Further degradation of these metabolites, including fucose, sialic acid, and lactose through complex microbial interactions, drives structural and functional diversification of the gut microbiota. Notably, HMO-derived metabolites play a critical role in infant-microbe crosstalk, selectively enriching taxa associated with immune and metabolic health while helping exclude potential pathogens.^68^ Together, these findings establish HMO metabolites as key mediators that extend the influence of HMOs beyond the initial degradation by pioneer taxa, thereby broadly supporting microbiota assembly.

HMO metabolites critically drive microbiota assembly by mediating cross-feeding interactions within the gut microbiota. Through enzymatic hydrolysis or incidental processes such as cell lysis, HMO-derived end-products and by-products are released extracellularly.^69^ These metabolites, as public goods, fuel a cross-feeding network whose metabolic cascade through secondary and tertiary consumers structures the microbial community, fostering interactions that range from cooperative to competitive.^70^ A well-characterized paradigm of this cross-feeding occurs between *B. bifidum* and *B. breve*, which compete for one substrate but cross-feed on another.^67^ Specifically, as a primary degrader, *B. bifidum* extracellularly hydrolyzes 2'-FL, releasing lactose and fucose. While lactose is metabolized by *B. bifidum* itself, the liberated fucose is utilized by *B. breve*, which lacks extracellular fucosidases but harbors specialized *fuc* gene clusters for fucose assimilation. Although *B. breve* competes with *B. bifidum* for lactose, stable coexistence is maintained since the benefits of cross-feeding offset this competition; rapid fucose uptake by *B. breve* alleviates feedback inhibition on extracellular fucosidases, enabling continuous hydrolysis of 2'-FL by *B. bifidum*. This functional reciprocity establishes a stable ecological niche for both species, facilitating their co-colonization and dominance in the infant gut.

HMO-cross-feeding network exhibits considerable metabolic depth. Metabolites generated by *B. breve* from cross-fed fucose—notably lactate and 1,2-propanediol (1,2-PD)—support butyrate production in *Eubacterium hallii* and propionate generation in *Veillonella* spp.^42,^^71^This metabolic network also possesses broad taxonomic inclusivity, with taxa beyond *Bifidobacterium* such as *Lactobacillus* and *Ruminococcus* actively utilizing distinct HMO-derived metabolites.^48^^,^^72^ Thus, the provisioning of nutritional resources for a diverse array of commensals directly enhances the complexity and resilience of the assembled microbial community. Notably, however, HMO metabolites can also be exploited by potential pathogens. For instance, sialic acid released from milk oligosaccharides or host glycans promotes the expansion of enteric pathogens such as *Escherichia coli* and *Clostridium difficile.*^73^ This indicates that microbial community assembly is a nutrient-driven process where the accessibility of liberated sugars to pathogens is highly context-dependent. This context-dependent risk is shaped by the diversity and metabolic competitiveness of the resident microbiota, as well as the local metabolic environment. A diverse and stable microbiota establishes colonization resistance via mechanisms such as priority effects and nutrient blocking, thereby limiting the nutrient niche for pathogens.^74^ Conversely, in states of ecological perturbation including post-antibiotic or inflamed dysbiosis, the depletion of protective taxa compromises colonization resistance. Under these conditions, the resulting increase in liberated sialic acid can facilitate expansion of opportunistic pathogens such as *Salmonella typhimurium.*^73^ Therefore, quantifying the effect sizes of HMO metabolism across these diverse contexts, from baseline diet to states of dysbiosis, will be essential to guide context-specific nutritional strategies.

In addition to sustaining cross-feeding networks, HMO metabolites directly modulate microbial colonization by selectively altering microbial fitness in the gut. This is exemplified by SCFAs such as acetate, which lowers intestinal pH to inhibit pH-sensitive pathogens while favoring butyrate-producing *Roseburia.*^75^ Apart from such physicochemical means, HMO metabolites can directly interfere with microbial virulence. For example, host-derived fucose represses biofilm-related genes and formation in opportunistic pathogens, thereby compromising their ability to colonize the colonic crypts.^76^ In contrast, the same fucose molecule can be incorporated by commensal *Bacteroides* into capsular polysaccharides and glycoproteins, components that are critical for competitive gut colonization.^58^ Consequently, by simultaneously suppressing pathogens and selecting for commensals, HMO metabolites emerge as central drivers of beneficial microbiota assembly during infancy.

In summary, HMO metabolites are established drivers of infant gut assembly by fueling cross-feeding networks and directly modulating microbial fitness. While model systems continue to uncover metabolites beyond SCFAs—such as indole-3-lactic acid, which modulates early-life immunity^77^—a direct causal link between these metabolites, HMO metabolism, and specific infant physiology is still lacking. Future research should therefore bridge this gap and quantify the ecological impact of the entire HMO metabolome across diverse infant contexts.

### 4.2. Signaling modulation via HMOs

In most cases, 40%−50% of ingested HMOs bypass intestinal catabolism in breastfed infants, with structurally intact forms consistently detected in their feces.^14^ This suggests HMOs mediate biological functions transcending their role as prebiotic nutrients. Specific HMOs are increasingly recognized as signaling molecules that, acting independently of their metabolic breakdown, directly engage host or microbial receptors or act as molecular decoys to regulate adhesion and immune responses. In this section, we synthesize current evidence on how HMOs, functioning as signaling molecules, promote commensal colonization by directly modulating microbial adhesion and shaping a symbiont-favorable host immune microenvironment (Figure 1). Evidence supporting these signaling functions arises from distinct experimental tiers: biophysical and pathogen adhesion assays demonstrating decoy binding; cell culture and murine models showing HMO-induced epithelial glycosylation changes; and animal and cellular studies implicating immune receptors. In human infants, however, the direct causal pathways linking specific HMO structures to their cognate receptors and functional outcomes remain to be mapped.

#### 4.2.1. HMOs-mediated microbial adhesion

Adhesion at the host-microbe interface is a critical initial step for microbial colonization, where HMOs exert exquisite selectivity. Through structural mimicry of epithelial receptors and modulation of host mucin glycocalyx, HMOs shape a symbiont-promotive niche while enforcing competitive exclusion of potential pathogens. Key commensals, including *B. infantis, B. bifidum* and *A. muciniphila*, have been demonstrated to enhance their adhesion to intestinal epithelial cells through HMO-induced upregulation of adhesive factors, such as fibronectin-binding autotranslator adhesives, sortase-dependent pili, and moonlighting proteins.^46^^,^^78^^,^^79^ However, this pro-adhesive effect appears to be primarily a consequence of utilizing HMOs as growth substrates, as indicated by the concurrent upregulation of HMO-catabolizing gene clusters in these bacteria.^46^^,^^78^ Limited evidence currently supports the existence of a direct HMO-triggered signaling pathway for symbiont adhesion independent of metabolic catabolism.

In contrast, HMOs function as signaling mediators that directly interfere with pathogen adhesion. Their structural resemblance to epithelial glycans allows them to act as soluble decoys, which competitively inhibit pathogenic binding to host receptors.^80^ This anti-adhesive function has been extensively documented against a range of intestinal pathogens, including *Campylobacter jejuni*, *Clostridium difficile*, *Escherichia coli*, and *Salmonella fyris.*^81^^,^^82^ The mechanism is rooted in structure-specific molecular recognition: particular glycan motifs— such as fucosyl and sialyl residues—selectively engage with pathogen adhesins to block host-pathogen interactions. For instance, *C. jejuni* relies on recognizing the intestinal H-2 antigen (Fucα1,2Galβ1,4GlcNAc) for mucosal colonization.^81^ α1,2-fucosylated HMOs, which share the Fucα1,2 Gal motifs, competitively occupy these host epitopes, thereby hindering epithelial attachment and decreasing both cellular infectivity *in vitro* and clinical diarrheal incidence. Although the structural basis remains incompletely resolved, recent work suggests that *C. jejuni* may bind fucosylated HMOs via lectin-like adhesins, as indicated by its specific interaction with fucoidan.^83^ Similarly, fucosylated oligosaccharides inhibit certain *Escherichia coli* strains by binding to guanylate cyclase and disruption of enterotoxin activity and adhesion.^84^ Other HMO structures also exhibit competitive inhibition: Lacto-*N*-fucopentaose V (LNFPV) and Lacto-*N*-neohexaose (LNnH) suppress *Clostridioides difficile* toxin A (TcdA) by high-affinity occupying its carbohydrate-binding pocket in TcdA-f2,^85^ while 3'-SL mimics α2-3-sialylated host receptors to block *H. pylori* adhesion.^86^^,^^87^ Notably, this decoy function is transient and nonspecific, providing broad-spectrum protection against diverse pathogens—including enteric viruses (e.g. rotavirus) and fungi (e.g., *Candida albicans*) in addition to bacteria.^88–90^ Through this filtering of microbial exposure, HMOs help balance commensal colonization and pathogen exclusion during immune-immature gut microbiota assembly.

Apart from binding bacterial adhesins, HMOs selectively regulate microbial adhesion via engaging host epithelial cells to remodel the intestinal glycocalyx–the structured epithelial glycan layer that serves as the primary adhesion site for commensals.^61^ During postnatal development, the surface glycosylation pattern of intestinal epithelium undergoes marked changes, transitioning from sialylation to fucosylation predominance throughout the weaning transition.^91^ This shift coincides with the critical period of microbial colonization, implicating that host glycan remodeling actively shapes microbiota assembly.^76^ Extensive studies delineate how specific HMO direct glycocalyx remodeling. For instance, 2'-FL and LNnT enhance mucin synthesis and secretion through transcriptional activation of *MUC* genes in goblet cells, thereby increasing the epithelial surface area available for glycocalyx deposition.^46^^,^^92^ Meanwhile, 3'-SL and 2'-FL modulate the terminal glycan repertoire of the glycocalyx through regulating key glycosyltransferases in colonic epithelium, such as *FUT2*-catalyzed α1,2-fucosylation and *ST3GAL1/2/4*-mediated sialylation.^93^^,^^94^ These HMO-modified fucosylated and sialylated glycans provide critical binding targets for bifidobacterial moonlighting proteins AfcA and SiaBb2, which function as recognition adhesins rather than catalytic enzymes to specifically bind host cognate conjugates and promote colonization.^50^^,^^95^ In parallel, 2'-FL-induced mucin glycosylation creates a favorable niche for mucin-utilizing taxa such as *Akkermansia* and *Bacteroides* by supplying their preferred glycan substrates.^46^^,^^93^ This glycan remodeling also reinforces a pathogen exclusion barrier: 3'-SL-mediated attenuation of α2,3- and α2,6-sialylation epitopes reduce ETEC adherence by approximately 50%.^94^ Importantly, increased microbial adhesion or persistence does not necessarily translate into host benefit. Although mucin-associated colonization enhances microbial fitness by improving nutrient access and niche retention, excessive adhesion, rapid mucin degradation, or crypt occupation can compromise the mucus barrier, increase epithelial exposure, and promote low-grade inflammation—particularly in hosts with immature immunity or pre-existing epithelial stress.^73^ Therefore, HMO-mediated regulation of adhesion should not be viewed simply as promoting microbial persistence, but rather as a mechanism that limits overcolonization while maintaining mucus barrier integrity. This distinction underscores the need to separate microbial colonization advantages from host benefit, and to quantify the thresholds beyond which mucophilic foraging becomes detrimental to the infant host.

While studies primarily conducted *in vitro* or in murine models have established HMOs as modulators of mucin glycosylation, direct, catabolism-independent adhesion signaling remains to be demonstrated in human infant systems. Furthermore, although IL-22RA1 and TLR signaling is known to induce FUT2 expression and epithelial fucosylation,^96^^,^^97^ it is still unknown whether HMOs act through these pathways or activate distinct signaling cascades to regulate glycosylation.

Emerging multi-omics technologies and high-throughput interaction screening platforms offer promising avenues to address these questions.^15^ For example, integration of Microbiome Cartography spatial profiling, including MIBI spatial imaging, GeoMx DSP regional transcriptomics, and MALDI-MSI glycan analysis, with BASEHIT bacterial interaction mapping in HMO-treated neonatal murine models could systematically characterize how specific HMOs bind to host extracellular proteins and their resulting microbe-host crosstalk. Moving forward, a multi-scale investigative approach—encompassing animal models, *in vitro* systems, machine learning-based receptor prediction, and human observational studies tracking HMO-related glycoprotein modifications— will be essential to fully elucidate the mechanisms by which HMOs regulate microbial adhesion and colonization.

#### 4.2.2. HMO-mediated IgA regulation of microbiota assembly

The mucosal immune system plays a fundamental role in shaping microbial assembly and homeostasis during postnatal development, with secretory immunoglobulin A (sIgA) serving as a key mediator. SIgA promotes commensal colonization while excluding pathogen via glycan-dependent recognition; this process has been extensively reviewed elsewhere.^98^ Bacteria targeted by sIgA, particularly slow-growing, host-proximal niche-adapted species such as *Bacteroides fragilis*, *Bacteroides uniformis*, and *Akkermansia muciniphila*, often benefit from enhanced mucus adhesion and competitive colonization within mucosal niches via IgA coating.^99^^,^^100^ Notably, HMOs may indirectly modulate IgA-microbiota interactions through immune receptor-mediated regulation of IgA responses. It is well established that HMOs engage specific pattern recognition receptors (PRR) in immune and epithelial cells, notably including Toll-like receptors (TLRs) and lectin receptors, to coordinate immune signaling and mucosal homeostasis.^101^ Experimental evidence highlights the critical role of TLRs signaling in intestinal IgA production. For instance, transgenic mice with constitutive TLR4 activation in intestinal epithelial cells exhibit enhanced mucosal B-cell recruitment and elevated fecal IgA levels; this effect is abrogated by herpes virus M3 protein expression, further confirming TLR4-mediated control of intestinal IgA immunity.^102^ Mechanistically, TLR4 enhances IgA production through direct B-cell stimulation or via NF-κB-dependent induction of cytokines such as B cell–activating factor of the TNF family (BAFF) and a proliferation-inducing ligand (APRIL), which promote lamina propria B-cell recruitment and T-cell-independent IgA class switching.^102^ Collectively, these findings delineate an emerging but still inferred “HMOs-TLR-IgA” regulatory axis, through which maternal oligosaccharides are proposed to couple innate immune recognition to IgA-mediated microbial selection.

Converging evidence from model systems and randomized controlled trials indicates that HMO exposure is associated with IgA changes and microbiota shifts, yet the causal mechanisms underlying these associations in human infants remain incompletely defined. In mice, early-life exposure to dietary LPS induces intestinal germinal center reactions via TLR4-MyD88/TRIF signaling, promoting IgA^+^ plasma cell differentiation, somatic hypermutation, and IgA diversification independently of the microbiota.^103^ This glycan-driven, innate immune receptors-mediated IgA induction illustrates a plausible mechanism by which dietary molecules, including HMOs, modulate mucosal immunity. In human infants, supplementation of formula with five defined HMOs significantly increased fecal sIgA levels at 3-6 months, concomitant with a 45% enrichment in *B. longum* subsp*. infantis* and a notable 75−85% reduction in toxigenic *C. difficile.*^104^ However, given that fecal IgA in early infancy is predominantly of maternal origin, attributing its increase directly to HMO-driven endogenous production remains challenging.

In summary, while the engagement of TLR/PRR by HMOs and the IgA-inducing capacity of such signaling are well established, the existence of a direct “HMOs–TLR–IgA” pathway in human infants remains an inference from correlative evidence. Resolving this question is complicated by several confounding factors in early life. First, delayed maturation of the neonatal intestinal immune system (initiating at 4-8 weeks postpartum) results in intestinal reliance on human milk-derived sIgA during early development.^105^ Second, technical limitations preclude reliable discrimination between maternally derived and endogenously produced IgA in infants. Third, even as endogenous IgA production increases with immune maturation, this rise is primarily driven by microbiota-derived metabolites and extracellular vesicles.^106^^,^^107^

Notably, the challenge of defining HMO-immune causality extends beyond sIgA to other mucosal immune components, such as antimicrobial peptides (AMPs). Preliminary *in vitro* studies implicate specific HMOs in modulating the expression of human *β*-defensin-2 and defensin *β*-1.^108^^,^^109^ However, establishing a direct role for HMOs in AMP regulation *in vivo* faces even greater obstacles. Innovative approaches combining TLR conditional knockout models, targeted HMO receptor blockade, and longitudinal multi-omics profiling will be required to definitively clarify how HMOs mediate mucosal immunity and microbial community assembly.

Outstanding Questions

- **HMO-Receptor Specificity:** Which specific HMO structures engage which host receptors (e.g., TLRs, lectins) *in vivo*, and what are the downstream functional consequences?
- **Causal IgA Induction:** Can defined HMO mixes, combined with conditional receptor (e.g., TLR) knockout models, establish a direct, microbiota-independent causal pathway for IgA induction?
- **Tracking Endogenous IgA:** How can maternal and endogenous infant sIgA be reliably distinguished in longitudinal studies? Can glycoproteomic or IgA sequencing approaches resolve this?

## 5. Conclusions and outlook

HMOs critically govern early-life gut microbiota assembly by functioning as both metabolic substrates and signaling molecules. Their structural complexity imposes selective pressure that enriches specialized degraders harboring dedicated enzymatic machinery (e.g., NanH, PULs, MULs). The metabolites released by these organisms, in turn, fuel cross-feeding networks that drive the assembly of a diverse and stable microbial community. Crucially, as signaling molecules, HMOs modulate mucosal glycosylation, shape IgA responses, and act as soluble decoys. Through these multifaceted mechanisms, HMOs collectively optimize the gut environment for symbiotic colonization and mucosal defense.

Despite significant advances, key mechanistic questions remain. First, although enzymes such as NanH and PerB have been implicated in niche adaptation, the precise molecular determinants of how and why they confer a competitive fitness advantage through specific HMO-enzyme interactions remains unknown. Addressing this requires a shift from phenotypic observation to mechanistic dissection. This necessitates integrating machine learning-based genome-wide association studies (GWAS) for gene discovery,^110^ high-throughput mutagenesis for functional validation,^111^ and high-resolution structural biology to elucidate the molecular determinants of fitness. Second, the signaling functions of HMOs remain largely inferred, partly due to the difficulty of matching diverse HMO structures to specific receptors and the functional redundancy among signaling pathways. A combination of computational prediction and novel experimental methods—such as HMO-specific biosensors and biotin- or fluorescently-labeled probes— will be essential to systematically identify HMO-binding proteins from intestinal tissues and cellular membranes. Third, future research should strive to situate HMOs within a holistic ecological context by dissecting how they interact with other key determinants of microbial assembly, such as colonization order and host immunity, to collectively shape the infant gut microbiota. For example, studies on priority effects show that arrival order typically determines dominance in the absence of HMOs; however, HMO supplementation can override priority effects, enabling superior HMO utilizers like *B. longum* subsp. *infantis* to dominate regardless of arrival timing,^40^ an outcome contingent on microbial and HMO-specific factors. Collectively, addressing these key unknowns is essential. Moving forward, translating these model-derived insights into diverse human infant contexts, which vary in HMO exposure, microbial inocula, and host genetics, represents a critical frontier for developing targeted nutritional interventions.
